# In Silico Molecular Docking Analysis, Cytotoxicity, and Antibacterial Activities of Constituents of Fruits of *Cucumis dipsaceus*

**DOI:** 10.1021/acsomega.3c08866

**Published:** 2023-12-19

**Authors:** Teshale Assefa, Hailemichael Tesso, Venkatesha Perumal Ramachandran, Leta Guta, Taye B. Demissie, Japheth O. Ombito, Rajalakshmanan Eswaramoorthy, Yadessa Melaku

**Affiliations:** † Department of Applied Chemistry, Adama Science and Technology University, P.O. Box, Adama 1888, Ethiopia; ‡ Department of Applied Biology, Adama Science and Technology University, P.O. Box, Adama 1888, Ethiopia; § Department of Chemistry, University of Botswana, Gaborone P/Bag 00704, Botswana; ∥ Department of Biomaterials, S Aveetha Dental College and Hospitals, Saveetha Institute of Medical and Technical Sciences (SIMATS), Saveetha University, Chennai 600 077, India

## Abstract

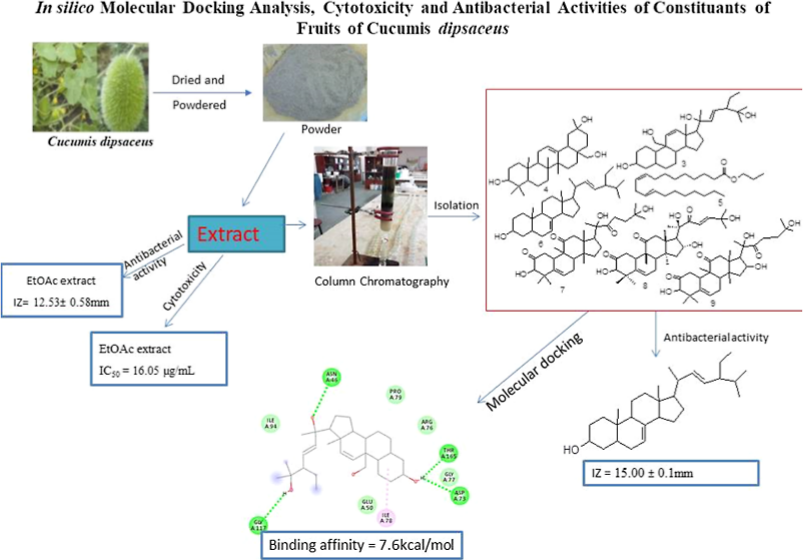

*Cucumis dipsaceus* (Cucurbitaceae) is a plant traditionally used against diarrhea, teeth-ach, wounds, stomach ache, meningitis, and cancer. The extracts of *C. dipsaceus* after silica gel column chromatography gave nine compounds identified using spectroscopic methods such as hexacosane (**1**), octadecane (**2**), 17-(-5-ethyl-2,6-dihydroxy-6-methylhept-3-en-2-yl)-9-(hydroxymethyl)-13-methylcyclopenta[α]phenanthren-3-ol (**3**), erythrodiol (**4**), (9,12)-propyl icosa-9,12-dienoate (**5**), α-spinasterol (**6**), 16-dehydroxycucurbitacin (**7**), cucurbitacin D (**8**), and 23,24-dihydroisocucurbitacin D (**9**). Compounds **3** and **4** are new to the genus *Cucumis*. α-Spinasterol showed better inhibition zone diameter = 13.67 ± 0.57, 15.00 ± 0.10, and 13.33 ± 0.57 mm against *Escherichia coli*, *Pseudomonas aeruginosa,* and *Streptococcus pyogenes* compared with the other tested samples. α-Spinasterol (−8.0 kcal/mol) and **3** (−7.6 kcal/mol) displayed high binding affinity against DNA Gyrase compared to ciprofloxacin (−7.3 kcal/mol). α-Spinasterol and 16-dehydroxycucurbitacin showed better binding affinity against protein kinase. The cytotoxicity results revealed that the EtOAc extract showed the highest potency with IC_50_ = 16.05 μg/mL. 16-Dehydroxycucurbitacin showed a higher binding affinity (−7.7 kcal/mol) against human topoisomerase IIβ than etoposide. The cytotoxicity and antibacterial activities and in silico molecular docking analysis displayed by the constituents corroborate the traditional use of the plant against bacteria and cancer.

## 1 Introduction

Infectious diseases caused by pathogenic microorganisms have become a major public concern globally. The current antibiotics available on the market are increasingly losing their effectiveness due to the accumulating mutations in bacterial species in addition to the development of resistance by pathogenic microorganisms.^1^ Several human ailments result from the occurrence of unstable free radicals in the human body that can mainly be alter the cell membranes and other structures such as proteins, lipoprotein, and DNA.^2^ Other morbidities such as cancer have highly contributed to several deaths around the world and continue to pose a constant threat.^3^ Plant extracts have been the major source of many therapeutics, including antimicrobial and anticancer agents due to their relative lack of toxicity as compared to their synthetic counterparts.^4^

The genus *Cucumis* (Cucurbitaceae) comprises 800 species distributed in Africa, Asia, and Australia. Some species are traditionally used against tetanus, tuberculosis, typhoid, cancer, and cholera.^5−7^ Pharmacological reporting showed that the extracts of *Cucumis* species had antibacterial, antihyperglycemic, antiviral, and anticancer activities.^8^*Cucumis dipsaceus* Ehrenb ex. Spach is an annual climbing and flowering plant distributed in various parts of Ethiopia.^9,10^*C. dipsaceus* is named hollotoo in Afan Oromo and Yemidir Embuway in Amaharic, Ethiopia. Traditionally, the leaves of *C. dipsaceus* are consumed by the local community as vegetable. The fruit is used for the treatment of gastrointestinal diseases, diarrhea, cancer, and meningitis.^11^ Several pharmacological activities, including hepatoprotective,^12^ analgesic,^13^ anti-inflammatory,^14^ cytotoxic,^15^ antioxidant,^16^ antimicrobial,^17^ antiallergic,^18^ anticarcinogenic,^19^ and antiobesity activities,^20^ have also been reported on the extracts of *C. dipsaceus*. Despite the traditional use of *C. dipsaceus* against various diseases in Ethiopia, the scientific reports that dwell on the antibacterial and anticancer activity of this plant is limited. In view of this, a preliminary study conducted on the extracts of this plant for its in vitro antibacterial and cytotoxic effects showed desirable activities. Therefore, we conducted a phytochemical investigation on the fruit extracts of *C. dipsaceus* to identify the active compounds responsible for the antibacterial activity and cytotoxicity of the extracts. The in silico molecular docking analysis and pharmacokinetic properties of the isolated compounds were also included in this report.

## 2 Materials and Methods

### 2.1 Plant Collection, Authentication, and Preparation

The fruits of *C. dipsaceus* were collected from Dera, Arsi, and Oromia, Ethiopia, in October 2021. The plant was authenticated by Mr. Melaku Wendafirash and deposited at the National Herbarium of Addis Ababa University with voucher specimen number CD002. The plant material was washed with distilled water and air-dried. The dried fruits were separately grounded into powder using a Milling machine. The samples were then stored in polyethylene bag in a refrigerator.

### 2.2 Extraction and Isolation

The air-dried ground fruits (500 g) were successively extracted with petroleum ether, EtOAc, and methanol at room temperature for 72 h. Each extract was filtered and concentrated at 40 °C on a rotary evaporator to afford their corresponding extracts. The petroleum ether extract (18.9 g) was adsorbed and subjected to silica gel column chromatography (CC) using increasing polarity of EtOAc in petroleum ether to afford 225 fractions, each of 10 mL. Fractions 39–70, eluted using petroleum ether/EtOAc (85:15), were mixed and rechromatographed by using petroleum ether/EtOAc (9:1) as the eluent to give 25 fractions, out of which fractions 8–10 were identified as compound **1** (28 mg), while fractions 14 and 15 were found to be compound **2** (15 mg). Fractions 161–206, eluted using petroleum ether/EtOAc (3:2), are combined together and rechromatographed using petroleum ether/EtOAc (65:35) as the eluent to afford 30 fractions from which fraction 24 gave compound **3** (35 mg). Fractions 217–222 were rechromatographed over silica gel using petroleum ether/EtOAc (2:3) as the eluent to afford 10 fractions of which fraction 4 gave compound **4** (20 mg).

The EtOAc extract (7.83 g) was also fractionated by silica gel (150 g) column chromatography using petroleum ether for packing. Elution was carried out with petroleum ether/EtOAc (9:1) and increasing polarity of EtOAc in petroleum ether as the mobile phase to afford 130 fractions, each 10 mL. Fractions 1–15, eluted using petroleum ether/EtOAc (85:15), were rechromatographed using petroleum ether/EtOAc (9:1) to afford 42 fractions of 10 mL each. Fraction 8 was identified as compound **5** (10 mg). Fractions 18–54, eluted using petroleum ether/EtOAc (4:1), were rechromatographed using petroleum ether/EtOAc (9:1) to give 73 fractions, among which fractions 11–31 were mixed and further purified to afford compound **6** (30 mg).

Fractions 101–130, eluted using petroleum ether/EtOAc (1:1), were combined and rechromatographed over silica gel using petroleum ether/EtOAc (6:4) to give 170 fractions (10 mL each) out of which fractions 85–106 were identified as a mixture of compounds **7** and **8** (60 mg). Compound **9** (20 mg) was obtained from fractions 145–154.

### 2.3 Antibacterial Activity

Antibacterial activity of the samples was evaluated using the disc diffusion method against two Gram-negative [*Escherichia coli* (ATCC-25922)] and *Pseudomonas aeruginosa* [(ATCC-27853)] and two Gram-positive [*Staphylococcus aureus* (ATCC-25923)] and *Streptococcus pyogenes* [(ATCC-19615)] bacteria. Appropriate colonies of bacterial strains were standardized with 0.5 McFarland standards turbidity.^21^ Centrifuged pellets of bacteria from a 24 h old culture containing 1.5 × 10^8^ CFU (colony forming unit) per mL were spread on the surface of the nutrient agar which was autoclaved at 121 °C for 15 min and then cooled to 45 °C and 25 mL of this media poured into each Petri dishes and allowed to settle. Samples were prepared at concentrations of 400, 200, 100, and 50 μg/mL for extract and 100, 50, 25, and 12.5 μg/mL for isolated compounds in dimethyl sulfoxide (DMSO). After the solidification of media, the test samples and ciprofloxacin were loaded on to each disc. DMSO was used as the negative control. All plates were observed for a zone of inhibition at 35 °C after 16–18 h. The activity was then determined by measuring the inhibition zone diameter (IZD) in mm. The samples were analyzed in triplicates and the results were presented as M ± SD.

### 2.4 Determination of the MIC

The minimum inhibitory concentration (MIC) of the antimicrobials was determined by the method involving microdilution in culture broth, as indicated by the Clinical and Laboratory Standards Institute (CLSI) of the USA.^22^ MIC is the lowest concentration of an antimicrobial that inhibits the visible growth of a microorganism after 24 h incubation. Stock solutions of standard compound and isolated compounds were prepared in 2 mL of DMSO. This stock solution with different concentration such as 100, 50, 25, 12.5, 6.25, 3.12, and 1.56 μg/mL were prepared. 0.2 μL culture bacterial strains standardized with 0.5 McFarland standards turbidity were added to each test tube nutrient agar which was autoclaved at 121 °C for 15 min then cooled to 45 °C and incubated for 24 h at 37 °C and the growth was monitored visually and spectrophotometrically. The lowest concentration (highest dilution) required to arrest the growth of bacteria was regarded as the MIC.

#### 2.4.1 Determination of the MBC

The minimum bactericidal concentration (MBC) for the antimicrobials was determined using the dilution broth method.^22^ After 48 h of incubation at 37 °C, a volume of 0.1 mL was taken out of the wells in the Petri dish plates where no growth was seen, and it was subsequently injected onto the surface of the plates. MBC was determined to be the lowest concentration of the drug at which no colonies developed under these conditions, and they were cultured for 48 h at 37 °C. The absence of any growth on a Petri dish plate suggested that the concentration was below the technique’s limit of detection, which is 10 CFU/mL. As a result, the starting concentration of 10^5^ CFU/mL had dropped to less than 10 CFU/mL. As a result, the MBC was determined to be the lowest antimicrobial concentration that could effectively inactivate over 99.99% of the bacteria that were present. For every strain and antibacterial agent, three duplicates were carried out.

### 2.5 Cytotoxic Activity

Human breast cancer cell line MCF-7 was used to examine the potential cytotoxicity of the extracts of *C. dipsaceus*. The National Centre for Cell Science (NCCS) in Pune, India, provided the human breast cancer cell line MCF-7. The cells were cultured in T25 culture flasks with DMEM supplemented with 10% FBS and 1% antibiotics (100 U ml^–1^ penicillin and 100 μg mL^–1^ of streptomycin). The cells were kept at 37 °C in an environment that was humidified and contained 5% CO_2_. The cells were trypsinized and passaged for use in a subsequent experiment once they had reached confluency. In human breast cancer cell lines, the samples antiproliferative effects were investigated at modest concentrations (20 μg). Confluent cells were separated using trypsin–EDTA solution, and 5000 cells per well were used for culturing. At 50% confluence, the culture media was evacuated, and the cells were treated for 24 h at 37 °C in the CO_2_ incubator with 20 μg of plant extract in DMSO (20 μL) for 24 h at 37 °C in the CO_2_ incubator. Later cells were incubated with MTT (4 mg mL^–1^) for 3 h. With the use of a typical microplate reader, the absorbance was determined at 540 nm.^23^ The percent cytotoxicity (% cytotoxicity) was used to express the cell viability.^24^ The findings are shown as the average of three replicated experiments. The following equation was used to calculate the cell viability
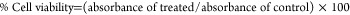


Using GraphPad Prism ver. 8.0, the values for the half-maximum inhibitory concentration (IC_50_) were calculated from a concentration–response curve of the percentage of cell viability (*y*-axis) versus log concentration (g/mL) of plant extract (*x*-axis).

### 2.6 Pharmacokinetic Studies of the Isolated Compounds

The test compounds were subjected to computational studies to predict their drug-likeness property, viz. Lipinski’s rule of five (ROF),^25^ Veber rule,^26^ and pharmacokinetic (ADME) properties using SwissADME^27^ and pkCSM^28^ online tools. The online program ProTox-II^29^ was used to predict the toxicity of the test and standard drugs.

### 2.7 Molecular Docking Analysis

The interaction and the binding affinity of the isolated compounds were investigated against *E. coli* (PDBID: 6F86), *S. aureus pyruvate kinase* (PDB ID: 3T07), and human topoisomerase IIβ (PDB ID: 3QX3). The crystal structures of the proteins were downloaded from the protein database and processed by removing the cocrystallized ligands, deleting water molecules, and adding polar hydrogen and cofactors according to the AutoDock 4.2 (MGL tools1.5.7) procedure. After the protein was cleaned, only polar hydrogens and the Kollman charges were introduced. In line with the experiment, we used ciprofloxacin as a control and standard drug. The grid center coordinates were 70, 70, and 70 pointing in the *x*, *y*, and *z* directions, respectively, with a grid point spacing of 0.375 Å. The center grid boxes were 9.393, −0.025, and 13.018 Å. 50 different conformations were generated for each targeted isolated compound. The conformation of the free compounds with the lowest free binding energy was selected to analyze the interactions with the receptors by using the Discovery Studio Visualizer.

## 3 Results and Discussion

**Figure 1:**
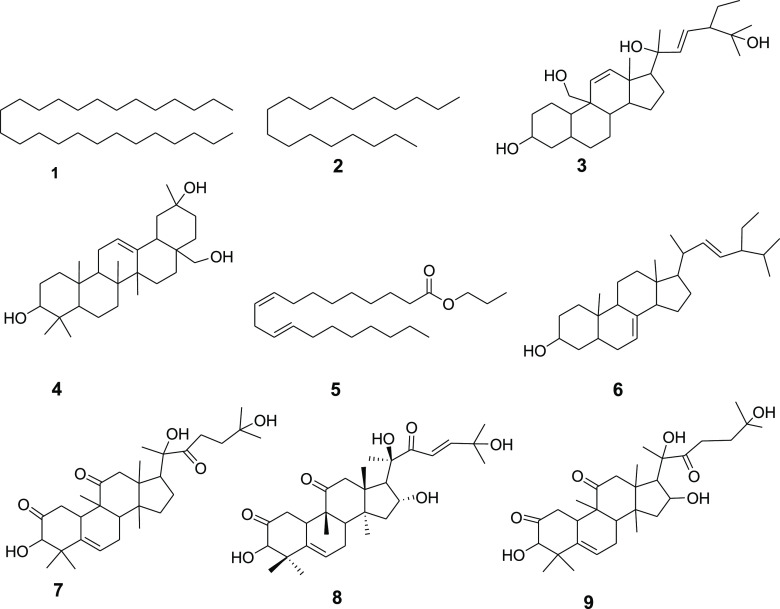
Structure of compounds isolated from the fruits of *C. dipsaceus*.

Nine compounds (Figure 1) were isolated and characterized from the fruit extracts of *C. dipsaceus*. The details are presented as follows.

Compound **1** was isolated as a white solid melting at 56–57 °C [lit. 56 °C].^30^ TLC showed a spot at *R*_f_ 0.95 using petroleum ether/EtOAc (9:1). Based on ^1^H, ^13^C NMR, and DEPT-135 spectral data, compound **1** is identified as hexacosane (Figure 1). Compound **2** was isolated as a white solid melting at 27–28 °C [lit. 28 °C].^31^ The TLC profile showed a spot at *R*_f_ 0.93 using petroleum ether/EtOAc (9:1). The NMR spectral data of compound **2** is in agreement with octadecane (Figure 1). Compound **3** showed a spot on TLC at *R*_f_ 0.60 with petroleum ether/EtOAc (4:1). The spectroscopic data generated for compound **3** agreed well with 17-(-5-ethyl-2,6-dihydroxy-6-methylhept-3-en-2-yl)-9-(hydroxymethyl)-13-methylcyclopenta[α]phenanthren-3-ol (Figure 1). Compound **4** was isolated as a solid from the petroleum ether extract of the fruits of *C. dipsaceus*. The sample melts at 231–232 °C [lit. 230 °C].^32^ TLC showed a spot at *R*_f_ 0.64 with petroleum ether/EtOAc (4:1). Based on ^1^H, ^13^C NMR, and DEPT-135 spectral data and literature support, compound **4** is established as erythrodiol (Figure 1).^33^ Compound **5** was isolated as a solid from the EtOAc extract of the fruits of *C. dipsaceus*. TLC showed a spot at *R*_f_ 0.76 with petroleum ether/EtOAc (9:1). Based on ^1^H, ^13^C NMR, and DEPT-135 spectral data, compound **5** is (9,12)-propyl icosa-9,12-dienoate (Figure 1). Compound **6** was isolated as a solid from the EtOAc extract of the fruit of *C. dipsaceus* melting at 170–171 °C [lit. 169–171].^34^ The TLC showed a spot at *R*_f_ 0.61 with petroleum ether/EtOAc (4:1). Based on ^1^H, ^13^C NMR, and DEPT-135 spectral data and comparison with literature value, compound **6** is in agreement with α-spinasterol (Figure 1).^34^

Compound **7** was isolated as a solid from the EtOAc extract of the fruits of *C. dipsaceus*. Its TLC showed a spot at *R*_f_ 0.36 with petroleum ether/EtOAc (3:2). Based on ^1^H, ^13^C NMR, and DEPT-135 spectral data and comparison with literature value, compound **7** is in good agreement with 16-dehydroxycucurbitacin D (Figure 1).^35^

Compound **8** was isolated as a solid melting at 150–151 °C [lit. 151.2 °C].^36^ Its TLC showed a spot at *R*_f_ 036 with petroleum ether/EtOAc (6:4) was used as a mobile phase. Based on ^1^H, ^13^C NMR, and DEPT-135 spectral data and comparison with literature value, compound **8** is in good agreement with cucurbitacin D (Figure 1).^37^

Compound **9** (20.1 mg) was isolated as a white solid from ethyl acetate extract of the fruits of *C. dipsaceus*. Its TLC showed a spot at *R*_f_ 0.22 with *n*-hexane: EtOAc (6:4) as a mobile phase. Based on ^1^H, ^13^C NMR, and DEPT-135 spectral data and comparison with literature value, compound 9 is in good agreement with 23,24-dihydroisocucurbitacin D (Figure 1).^37^

### 3.1 Antibacterial Activity

The antibacterial activity of the constituents was assessed using the agar disc diffusion method against *E. coli*, *P. aeruginosa*, *S. aureus,* and *S. pyogenes*. The results are presented in Table 1.

**Table 1:** Antibacterial Activity of Fruit Extracts and Isolated Compounds **1–9** of *C. dipsaceus*^a^

		IZD (mm)			
samples	conc. (μg/mL)	E. coli	P. aeruginosa	S. aureus	*S. pyogenes*
petroleum ether extract	400	11.0 ± 0.20	12.33 ± 0.52	10.67 ± 0.58	13.00 ± 0.20
	200	10.67 ± 0.58	10.67 ± 0.31	10.00 ± 0.2	12.00 ± 0.73
	100	9.67 ± 0.76	9.83 ± 0.56	9.83 ± 1.04	11.17 ± 0.61
	50	9.00 ± 0.00	7.67 ± 0.15	9.50 ± 0.50	10.50 ± 0.50
EtOAc extract	400	12.53 ± 0.58	11.87 ± 0.40	12.40 ± 0.15	9.50 ± 0.56
	200	11.93 ± 0.12	10.53 ± 1.30	11.20 ± 0.80	9.13 ± 0.10
	100	9.93 ± 0.38	9.83 ± 0.29	10.33 ± 1.80	8.60 ± 0.72
	50	8.53 ± 0.58	9.57 ± 0.40	10.20 ± 1.15	7.50 ± 0.56
MeOH extract	400	8.33 ± 0.57	8.0 ± 1.0	8.16 ± 0.28	7.10 ± 0.10
	200	7.40 ± 0.52	7.33 ± 0.61	7.13 ± 0.11	6.83 ± 0.28
	100	7.33 ± 1.04	6.83 ± 0.28	7.00 ± 0.0	6.66 ± 0.28
	50	6.33 ± 0.15	6.4 ± 0.17	6.60 ± 0.28	6.43 ± 0.11
1	100	8.67 ± 0.50	8.10 ± 0	6.13 ± 0.05	7.00 ± 1.0
	50	8.50 ± 0.45	7.0 ± 1.0	6.10 ± 0.0	6.90 ± 0.65
	25	8.00 ± 0.54	6.57 ± 0.4		6.57 ± 0.40
	12.5	7.80 ± 0.57	6.33 ± 0.20		6.33 ± 0.20
2	100	9.40 ± 0.17	7.83 ± 0.28	_	6.46 ± 0.05
	50	9.33 ± 0.11	7.33 ± 0.11	_	6.83 ± 0.28
	25	8.27 ± 0.05	7.16 ± 0.06	_	6.43 ± 0.35
	12.5	6.10 ± 0.00	6.45 ± 0.3	_	6.20 ± 0.10
3	100	13.0 ± 0.5	12.00 ± 0.20	6.20 ± 0.0	12.45 ± 0.52
	50	12.83 ± 0.76	11.33 ± 0.2	6.10 ± 0.0	11.33 ± 0.30
	25	10.50 ± 0.50	10.33 ± 0.54		10.00 ± 1.00
	12.5	9.00 ± 1.00	9.7 ± 0.60		9.00 ± 1.00
4	100	9.30 ± 0.99	10.50 ± 0.70	_	6.37 ± 0.15
	50	8.50 ± 0.32	9.60 ± 0.9		6.57 ± 0.40
	25	7.50 ± 0.50	9.4 ± 0.06	_	6.33 ± 1.0
	12.5	7.20 ± 0.03	7.80 ± 0.24	_	_
5	100	10.90 ± 0.71	11.60 ± 0.82	6.13 ± 0.05	7.33 ± 0.52
	50	10.50 ± 0.05	10.5 ± 0.03	6.10 ± 0.0	6.10 ± 0.10
	25	9.70 ± 0.80	8.50 ± 0.45	6.10 ± 0.0	6.57 ± 0.40
	12.5	7.20 ± 0.80	7.00 ± 0.45	_	6.33 ± 0.20
6	100	13.67 ± 0.57	15.0 ± 0.10	7.40 ± 0.10	13.33 ± 0.50
	50	11.67 ± 0.55	14.33 ± 0.52	7.23 ± 0.65	12.67 ± 0.57
	25	11.33 ± 0.34	12.0 ± 0.25	7.03 ± 0.15	11.00 ± 0.50
	12.5	10.67 ± 0.57	11.0 ± 1.0	6.50 ± 0.05	10.50 ± 0.50
7 and 8	100	10.50 ± 0.32	9.6 ± 0.52	6.23 ± 0.15	7.17 ± 0.76
	50	9.26 ± 0.64	8.5 ± 0.23	6.15 ± 0.10	6.57 ± 0.40
	25	8.00 ± 0.50	7.50 ± 0.65	6.10 ± 0.0	6.37 ± 0.15
	12.5	7.50 ± 0.50	7.00 ± 0.45		
9	100	11.50 ± 0.71	12.60 ± 0.82	6.13 ± 0.05	8.33 ± 0.52
	50	10.50 ± 0.05	10.5 ± 0.03	6.10 ± 0.0	6.10 ± 0.10
	25	9.70 ± 0.80	7.50 ± 0.45	6.10 ± 0.0	6.57 ± 0.40
	12.5	8.20 ± 0.80	7.00 ± 0.45	_	6.33 ± 0.20
Ciprofloxacin	30	16.63 ± 1.55	15.8 ± 1.15	15.65 ± 2.27	16.33 ± 1.65
	50	18.63 ± 0.55	17.8 ± 0.15	17.65 ± 0.27	18.33 ± 0.65

a The experiments were done in triplicates and results were presented as mean ± SD.

As revealed in Table 1, the extracts displayed a broad range of antibacterial activity against all pathogens in a dose-dependent manner. The petroleum ether extract exhibited the highest activity against *S. pyogenes* and *P. aeruginosa* with IZDs of 13.00 ± 0.20 mm and 12.33 ± 0.52 mm at 400 μg/mL, while the EtOAc extract displayed activity against *E. coli* and *S. aureus* with IZDs of 12.53 ± 0.58 mm and 12.4 ± 0.15 mm at 400 μg/mL, respectively. Compounds **1**–**9** displayed a wide range of antibacterial activity with inhibition zones ranging from 6.10 ± 0.0 to 15.00 ± 0.10 mm. Compound **6** displayed IZDs of 13.67 ± 0.57, 15.00 ± 0.10, and 13.33 ± 0.57 mm against *E. coli*, *P. aeruginosa,* and *S. pyogenes* at 100 μg/mL, respectively. The activity is significant compared with ciprofloxacin. At the same concentration, compound **3** exhibited IZDs of 13.0 ± 0.5, 12.00 ± 0.20, and 12.45 ± 0.52 mm against *E. coli*, *P. aeruginosa,* and *S. pyogenes*, respectively. Compound **9** had IZDs of 11.50 ± 0.71 and 12.60 ± 0.82 mm at 100 μg/mL against *E. coli* and *P. aeruginosa*, respectively.

#### 3.1.1 Determination of the MIC

In the present study, the MIC of the isolated compounds was determined by using a serial dilution technique. The results obtained revealed that compound 6 showed highest activity against tested microorganisms (Table 2). The results obtained demonstrated that the in vitro antibacterial activity, in silico molecular docking analysis, and the MIC are in good agreement. This indicates that compounds 3, 6, and 9 are promising for further analysis as an antibacterial agent.

**Table 2:** MIC (μg/mL) Values of Isolated Compounds^a^

	bacterial species			
compounds	*E. coli*	*P. aeruginosa*	*S. aureus*	*S. pyogenes*
1	6.25	12.5	50.0	12.5
2	12.5	12.5		12.5
3	3.12	3.12	50.0	3.12
4	12.5	12.5		25.0
5	3.12	3.12	25.0	12.5
6	1.56	1.56	6.25	1.56
7	6.25	6.25	25.0	25.0
8	6.25	6.25	25.0	25.0
9	3.12	3.12	25.0	12.5
ciprofloxacin	0.78	0.78	0.78	0.78

a Indicates bacteria are resistant to the compounds >100 μg/mL; MIC (μg/mL) = minimum inhibitory concentration, that is lowest concentration to completely inhibit bacterial growth.

### 3.2 Cytotoxic Activity

The MTT assay is a quick, sensitive, quantitative, and reliable assay that is used to measure cell viability. The assay is based on the capacity of the cellular mitochondrial dehydrogenase enzyme in living cells to reduce the yellow water-soluble substrate 3-(4,5-dimethylthiazol-2yl)-2,5-diphenyl tetrazolium bromide (MTT) into a dark blue/purple formazan product, which is insoluble in water. To investigate anticancer activity, MCF-7 breast cancer cell lines were treated with the petroleum ether, EtOAc, and methanol extracts of the fruits of *C. dipsaceus* at concentrations 20 μg/mL, and their viability was evaluated by the MTT assay. The percent cell viability of the petroleum ether, EtOAc, and methanol extract of the fruits were found to be 81.86 ± 3.04, 59.12 ± 3.70, and 48.36 ± 2.48%, respectively. According to the American National Cancer Institute, a sample is active if its IC_50_ values are less than 30 μg/mL after an exposure time of 72 h in a preliminary MTT.^38^ The results in the present study revealed that the methanol, EtOAc, and petroleum ether extracts of fruits of *C. dipsaceus* induced a significant reduction of cell growth in breast cancer cell lines with IC_50_ = 16.81, 16.05, and 17.48 μg/mL, respectively. The results are close to those of doxorubicin with an IC_50_ value of 6.18 μg/mL. This indicates that the extracts of *C. dipsaceus* had significant cytotoxic activity.

### 3.3 Drug-Likeness and Pharmacokinetic Studies of the Isolated Compounds

Lipinski’s rule of five is one of the most effective tools for predicting new chemical entities’ drug-likeness.^25^ Except for compound **3** and the standard drug, all the test compounds showed one violation due to the high molecular weight (MW > 500) or lipophilic character (*c* Log *P* > 5). According to Veber’s rule,^26^ compounds **1**, **2**, and **5** had one violation because of more number of rotatable bonds (>10) as these compounds had open-chain structures (Table 3). Except for these compounds, all the test compounds and reference drug appeared to be good oral drug candidates based on these findings.

**Table 3:** Drug-Likeness Predictions of the Test Compounds Computed by SwissADME^a^

compound	formula	mol. wt.(g/mol)	NHD	NHA	Log *P* (*c* Log P)	Lipinski’s ROF violation	NRB	TPSA (Å^2^)	Veber’s rule violation
1	C_26_H_54_	366.71	0	0	10.09	1	23	0	1
2	C_18_H_38_	254.49	0	0	7.18	1	15	0	1
3	C_29_H_48_O_4_	460.69	4	4	4.45	0	6	80.92	0
4	C_29_H_48_O_3_	444.69	3	3	5.13	1	1	60.69	0
5	C_23_H_42_O_2_	350.58	0	2	7.15	1	19	26.3	1
6	C_29_H_48_O	412.69	1	1	6.89	1	5	20.23	0
7	C_30_H_44_O_7_	516.67	4	7	2.63	1	4	132.13	0
8	C_30_H_44_O_7_	516.67	4	7	2.63	1	4	132.13	0
9	C_30_H_46_O_7_	518.68	4	7	2.78	1	5	132.13	0
Cipro	C_17_H_18_FN_3_O_3_	331.34	2	6	1.1	0	3	74.57	0

a NHD = Number of Hydrogen donor; NHA = Number of Hydrogen acceptor; NRB = Number of rotatable bonds; and TPSA = total polar surface area.

### 3.4 Absorption, Distribution, Metabolism, Excretion, and Toxicity Studies

The reference drug and the test compounds exhibited high gastrointestinal (GI) absorption except for compounds **1**, **2**, **3**, and **5** (Table 4). The rate at which a substance penetrates the stratum corneum is measured by skin permeability (*K*_p_). This value is commonly used to quantify the transport of molecules in the epidermal skin’s outermost layer and to highlight the importance of skin absorption. The lesser the log *K*_p_ value, the lower the cutaneous permeability of the molecule.^39^ In this study, compared to ciprofloxacin, the test compounds showed a log *K*_p_ value (Table 4), so these compounds might have better skin permeation than ciprofloxacin. Except for compounds **2**, **5**, and **6**, all of the test compounds and CPFX are predicted to be P-gp substrates.

**Table 4:** ADME Predictions of the Test Compounds, Computed by SwissADME and pkCSM^a^

	Absorption			distribution		metabolism					excretion	
compound	GI ABS	log *K*_p_ cm/s	P-gp substrate	BBB permeability	log VD (L/kg)	CYP3A4 inhibitor	CYP2D6 inhibitor	CYP2C9 inhibitor	CYP2C19 inhibitor	CYP1A2 inhibitor	Log(CLtot)(log mL/min/kg)	OCT2 substrate
**1**	low	1.19	yes	1.128	0.312	no	no	no	no	no	2.071	no
**2**	low	–1.2	no	0.977	0.661	no	no	no	no	yes	1.924	no
**3**	high	–5.7	yes	–0.51	–0.254	no	no	no	no	no	0.662	no
**4**	high	–5.06	yes	–0.457	0.083	no	no	no	no	no	0.191	no
**5**	low	–2.18	no	0.801	0.19	no	no	no	no	yes	2.153	no
**6**	low	–2.92	no	0.811	0.007	no	no	no	no	no	0.611	no
**7**	high	–8.07	yes	–1.02	–0.245	yes	no	no	no	no	0.271	no
**8**	high	–8.07	yes	–0.996	–0.129	yes	no	no	no	no	0.271	no
**9**	high	–8.04	yes	–1.081	–0.228	yes	no	no	no	no	0.256	no
**cipro**	high	–9.09	yes	–0.587	–0.17	no	no	no	no	no	0.633	no

a GI = gastro-Intestinal, P-gp = P-glycoprotein, BBB = blood brain barrier, VD = volume of distribution, CYP = cytochrome-P, CLtot = total clearance, and OCT2 = organic cation transporter.

BBB permeability is one of the important parameters that molecules exhibit their action at CNS. Molecules with log BB > 0.3 are considered to readily cross the BBB, while molecules with log BB < −1 are poorly distributed to the brain.^28^ Compounds **1**, **2**, **5**, and **6** showed log BB > 0.3. So, these molecules might have readily crossed the BBB and act on the CNS, whereas other test compounds and ciprofloxacin might not readily cross the BBB. The volume of distribution (VD) is the theoretical volume that the total dose of a drug would be needed to be uniformly distributed to give the same concentration as in blood plasma. If log VD < −0.15, considered to be low; log VD > 0.45, is considered to be high.^28^ Compound **2** might have high VD, since it showed log VD > 0.45, whereas compounds **3**, **7**, **9**, and ciprofloxacin might have low VD, since these compounds showed log VD < −0.15.

About 60% of prescribed drugs are metabolized by CYP enzymes, with CYP3A4 accounting for about half of this metabolism, followed by CYP2D6, CYP2C9, and CYP2C19.^40^ None of the test compounds and ciprofloxacin showed inhibition for the enzymes CYP2D6, CYP2C9, and CYP2C19. Compounds **7**, **8**, and **9** exhibited inhibitions for CYP3A4, and compounds **2** and **5** for CYP1A2 (Table 4), suggesting that these enzymes may not have metabolized these compounds.

Drug’s total clearance (TC) is measured by the proportionality constant CLtot, and occurs primarily as a combination of hepatic and renal clearance. It is related to bioavailability and is important for determining dosing rates to achieve a steady-state concentration. The low value of logCLtot means a high drug half lifetime (*t*_1/2_).^28^ The CLtot values for compounds **4**–**9** were lower than those for ciprofloxacin, suggesting that these compounds could have high *t*_1/2_ values. Compounds **1**, **2**, and **5** had very high logCLtot values; however, compound **3** showed only a slight increase in the value over ciprofloxacin (Table 4). Organic cation transporter 2 (OCT2) is the key transporter for cation influx in the renal epithelial cells.^41^ None of the test compounds and ciprofloxacin displayed an OCT2 substrate (Table 4), suggesting that OCT2 may not be involved in the excretion of these compounds.

The level of toxicity is expressed on a scale from 1 to 6, with a greater number indicating a lower level of toxicity. It is determined based on the LD_50_ (mg/kg) value, which denotes a dose that will kill 50% of the test animal.^42^ Among all test samples, compounds **5** and **9** were expected to be the least or no toxic because they fell under class 6, whereas compounds **3** and **4** belong under class 5. Compound **6** displayed toxicity class 4, similar to ciprofloxacin, whereas compounds **1** and **2** displayed class 3, and compounds **7** and **8** appeared to be more toxic since they fell under class 2. None of the compounds showed hepatotoxicity, mutagenicity, or cytotoxicity. However, compounds **5**, **7**, **8**, and **9** showed cytotoxicity, and ciprofloxacin and all of the test compounds except three compounds (**1**, **2**, and **5**) showed immunotoxicity (Table 5).

**Table 5:** Toxicity Prediction of Test Compounds, Computed Using Pro-tox II

			toxicity				
compound	LD_50_ (mg/kg)	toxicity class	hepatotoxicity	carcinogenicity	immunotoxicity	mutagenicity	cytotoxicity
1	750	3	no	no	no	no	no
2	750	3	no	no	no	no	no
3	2340	5	no	no	yes	no	no
4	4300	5	no	no	yes	no	no
5	20000	6	no	yes	no	no	no
6	2000	4	no	no	yes	no	no
7	50	2	no	yes	yes	no	no
8	50	2	no	yes	yes	no	no
9	8800	6	no	yes	yes	no	no
Cipro	2000	4	no	no	yes	no	no

### 3.5 BOILED-Egg Model

**Figure 2:**
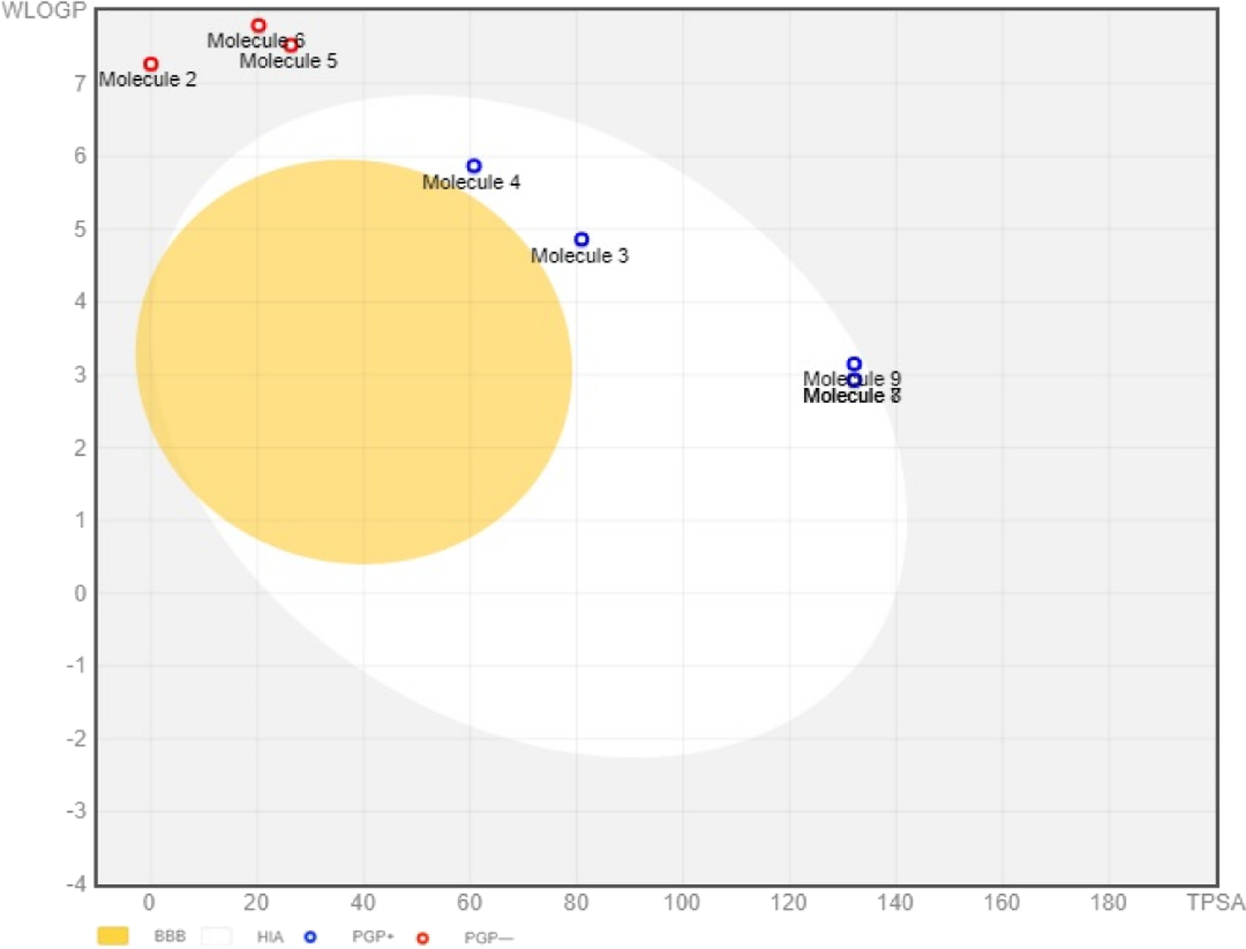
BOILED-Egg model for predicting gastrointestinal absorption and brain access. (B) Scale bar: 5 × 8 mm.

The cLogP and TPSA values of the compounds were plotted to predict human intestinal absorption (HIA) and blood–brain barrier (BBB) access (Figure 2). The egg-shaped plot is divided into three parts including a white area (HIA), a yellow area (BBB access), and a gray area (no HIA or BBB access).^43^ In this prediction, compounds **2**, **5**, and **6** were in the gray area, indicating no HIA or BBB access, whereas compounds **3**, **4**, **7**, **8**, and **9** were in the white area, indicating that these compounds may be absorbed through the intestine. None of the test compounds were in the yellow area, indicating that the test compounds might not have good BBB access. This model also predicted whether those compounds are substrates of P-gp (PGP) or not. Blue dots (PGP+) represent compounds that are substrates of PGP CNS efflux transporter and could be effluxed from the CNS, while red dots (PGP-) represent compounds that are not substrates of the PGP and could pass through and act on CNS.^43^ In this study, compounds **2**, **5**, and **6** had red dots, while others had blue dots, suggesting that only these compounds are not PGP substrates and eventually act on the CNS.

### 3.6 Molecular Docking Analysis of the Isolated Compounds against *E. coli* DNA Gyrase, *S. aureus*Pyruvate Kinase, and Human Topoisomerase IIβ

*E. coli* DNA gyrase is an enzyme that is required during bacterial DNA replication and transcription to maintain topology and integrity.^44^ Currently, DNA gyrase is considered one of the primary targets and has been clinically validated in most pathogenic bacteria.^45^ In this study, *E. coli* DNA gyrase (PDB ID: 6F86) was used as a target and a fluoroquinolone class of antibacterial, ciprofloxacin, was used as a reference control for docking with the target. Binding affinity and interactions with different amino acids are listed in Table 6.

**Table 6:** Binding Affinity and Interaction with the Target *E. coli* DNA Gyrase (PDB ID: 6F86)

			residual amino acid interactions	
compound	Δ*G*_binding_ (kcal/mol)	H-bond	hydrophobic/Pi-cation/Pi-anion/Pi-alkyl interactions	van-der Walls interactions
1	–4.8			
2	–4.7			
3	–7.6	Asn-46, Asp-73, Gly-117, Thr-165	Ile-78	Gly-77, Glu-50, Arg-76, Pro-79, Ile-94
4	–7.1	Asn-46	Ile-94	Glu-50, Pro-79, Arg-136, Ile-78
5	–5.1			
6	–8.0	Asp-73	Ile-94, Ile-78	Gly-77, Ala-47, Thr-165
7	–7.5	Asn-46, Asp-49, Arg-76, Val-93, Ile-94, Gly-119^a^, Pro-79^a^		Glu-50, Leu-98, Val-97
8	–7.0	Asn-46, Val-120, Ser-121, Leu-98		Gly-77, Glu-50, Arg-76, Ile-94, Gly-119, Leu-98
9	–7.0	Asn-46, Arg-76, Arg-136		Glu-50, Ile-94, Pro-79, Ile-78
Ciprofloxacin	–7.3	Asn-46, Asp-73, Asp-49^a^	Pro-79, Asn-46, Asp-49	Ala-47, Gly-77, Glu-50, Arg-76, Ile-78

a Carbon Hydrogen bond.

Among the test samples, compounds **6** (−8.0 kcal/mol), **3** (−7.6 kcal/mol), and **7** (−7.5 kcal/mol) showed higher binding affinity than ciprofloxacin (−7.3 kcal/mol). Compound 6, which has the highest binding affinity, also exhibited the highest efficacy (MIC = 1.56 μg/mL) among the test compounds in the in vitro tests. Compound 2 demonstrated the lowest binding affinity as well as the least efficacy in the in vitro test (MIC of 12.5 μg/mL). Ciprofloxacin formed hydrogen bonding interactions with Asn-46 and Asp-73. Similarly, compound **3** showed H-bonding interactions with Gly-117 and Thr-165. Other test compounds (**7**, **8**, and **9**) showed H-bonding interactions with Asn-46 along with other amino acids, whereas compounds **4** and **6** only with Asn-46 and Asp-73, respectively. Most of the compounds except **1**, **2**, and **5** exhibited van der Waals interactions with Glu-50 and Gly-77. The results of in silico molecular docking of isolated compounds are in agreement with in vitro antibacterial analysis, whereas the binding interactions of **3** to **9** with the target proteins are provided in the Supporting Information (Figures S1–S7).

Pyruvate kinase (PK) has been identified as a crucial enzyme in staphylococci and regulates their growth, antibiotic resistance, and biofilm formation.^46^ It was also found to be structurally distinct from human homologues, and hence, it provides a promising target for novel antimicrobial agents.^47^ For the docking studies, *S. aureus* PK (PDB ID: 3T07) was used as a target and ciprofloxacin was used as a reference control. Binding affinity and interactions with different amino acids are presented in Table 7.

**Table 7:** Binding Affinity and Interaction of Isolated Compounds with the Target *S. aureus* PK^a^

			residual amino acid interactions	
compound	affinity (kcal/mol)	H-bond	hydrophobic/Pi-cation/Pi-anion/Pi-alkyl interactions	van-der Walls interactions
1	–2.5			
2	–2.8			
3	–5.0	Ser-362, Asn-369	Leu-370	
4	–5.2	Ser-362	Ile-361, Ala-358	
5	–3.3			
6	–5.4	Ser-345	Ile-361, His-365	
7	–5.9	Ser-362, Asn-369, His-365		
8	–5.5	Ser-362, Thr-366, His-365*, Thr-353*		
9	–5.0	Ser-362, Thr-366, Thr-353, Ser-354		
Cipro	–4.9	Ser-362, Thr-366, Asn-369	Ile-361	His-365

a Carbon hydrogen bond.

Among the test and standard compounds 7 (−5.9 kcal/mol), **8** (−5.5 kcal/mol), **4** (−5.2 kcal/mol) **3**, and **9** (−5.0 kcal/mol) showed the better binding affinity, respectively, than ciprofloxacin (4.9 kcal/mol). Compound **6** exhibited superior binding affinity compared to the other test compounds, with the exception of compounds **7** and **8**, and had the highest efficacy in the in vitro test (MIC 6.25 μg/mL). With the exception of compound **1**, compound **2** showed the lowest binding affinity and lowest efficacy in the in vitro test (MIC of >100 μg/mL). Ciprofloxacin formed H-bonding interactions with Ser-362, Thr-366, and Asn-369. Compounds **3**, **4**, **7**, **8**, and **9** showed H-bonding interactions with one or two of these amino acids, except compound **6** showed interactions with Ser-345. Only compounds **4** and **6** showed residual amino acid interactions with Ile-361, similar to ciprofloxacin, whereas results of **3**–**9** are presented in the Supporting Information (Figures S8–14).

Topoisomerase II (TOP2) is a nuclear enzyme that catalysis the relaxing and unwinding of double-stranded DNA, which is essential for DNA operations like replication, transcription, and repair.^48^ Recent studies showed that TOP2β is an important target for many anticancer agents including etoposide (EVP).^49^ In this regard, the binding affinity of the isolated compounds was done against human topoisomerase, and the results are presented in Table 8.

**Table 8:** Binding Affinity and Interaction with Target Human Topoisomerase IIβ^a^

			residual amino acid interactions	
compound	affinity (kcal/mol)	H-bond	hydrophobic/Pi-cation/Pi-anion/Pi-alkyl interactions	van-der Walls interactions
1	–3.1			
2	–2.9			
3	–6.4	His-775, Asp-561, Glu-477	Ala-779	
4	–6.8	His-775, Asp-479		
5	–3.3			
6	–6.2	Asp-561	Arg-503	
7	–7.7	His-775, Gln-778, Arg-503*		
8	–7.2	His-775, Asp-561, Gly-776, Leu-502		
9	–6.4	His-775, Asp-561, Gly-776, Asp-479, Ser-480		
EVP	–7.5	His-775, Asp-561, Lys-505, Arg-503	Arg-503, His-775, Glu-522	Gly-504, Gly-776, Ala-779, Arg-729, Asp-559, His-774, Glu-477

a Carbon hydrogen bond.

Among the test compounds, only compound **7** showed higher binding affinity (−7.7 kcal/mol) than EVP (−7.5 kcal/mol), which showed H-bonding interactions with His-775, Asp-561, Lys-505, and Arg-503. Compounds **3**, **8**, and **9** showed similar H-bonding interactions with (His-775 and Asp-561) along with other amino acids, whereas 4 and **7** with His-775, and 6 only with Asp-561. Compound **6** showed residual interactions with Arg-503 which is similar to EVP, whereas the binding interactions of **3** to **9** with the target proteins were provided in the Supporting Information (Figures S15–21).

## 4 Conclusions

The petroleum ether and EtOAc extract of the fruits of *C. dipsaceus* afford nine (**1**–**9**) compounds. Among these, erthyrodiol and 17-(-5-ethyl-2,6-dihydroxy-6-methylhept-3-en-2-yl)-9-(hydroxymethyl)-13-methylcyclopenta[α]phenanthren-3-ol are new to the genus *Cucumis*. The extracts and isolated compounds displayed a broad range of antibacterial activity against tested bacteria with compounds 17-(-5-ethyl-2,6-dihydroxy-6-methylhept-3-en-2-yl)-9-(hydroxymethyl)-13-methylcyclopenta[α]phenanthren-3-ol (3), α-spinasterol (6), and dihydroisocucurbitacin D (9) displayed significant activities against *E. coli*, *P. aeruginosa,* and *S. pyogenes* comparable with ciprofloxacin. This was in good agreement with the MIC results. α-Spinasterol (−8.0 kcal/mol) and 16-dehydoxycucurbitacin D (−7.5 kcal/mol) displayed high binding affinity against *E. coli* DNA Gyrase compared with ciprofloxacin (−7.3 kcal/mol). The binding affinity of dihydroisocucurbitacin D was also comparable with that of the standard drug. α-Spinasterol and 16-dehydoxycucurbitacin D also showed better binding affinity compared with that of the standard against *S. aureus* protein kinase. The findings suggest that α-spinasterol, 16-dehydoxycucurbitacin, and dihydroisocucurbitacin D might be further explored as an antibacterial agent. The extracts of *C. dipsaceus* showed comparable cytotoxic activity with doxorubicin against the MCF-7 cell line, indicating the potential of the extracts as an anticancer agent. 16-dehydoxycucurbitacin showed higher binding affinity (−7.7 kcal/mol) against human topoisomerase IIβ. Most of the compounds had good ADME properties and also satisfy Lipinski’s rule of five. Therefore, the antibacterial and cytotoxic activities displayed by the constituents of *C. dipsaceus* corroborate the traditional use of this plant against bacteria and cancer.

## Supporting Information Available

2D and 3D interactions of the isolated compounds and standard drugs with the target proteins (PDF)

## Notes

The plant material was collected in accordance with the guideline suggested by Addis Ababa University. Furthermore, the fruits of *C. dipsaceus* was collected after getting written consent or permission from the local government.
