# Supplementary material for: In Silico Molecular Docking Analysis, Cytotoxicity, and Antibacterial Activities of Constituents of Fruits of Cucumis dipsaceus
Source: ACS Omega. 2023 Dec 19;9(1):1945–55. doi: 10.1021/acsomega.3c08866 (PMC10785779; doi:10.1021/acsomega.3c08866)

Supporting Information

In silico Molecular Docking Analysis, Cytotoxicity and Antibacterial Activities of Constituents of Fruits of Cucumis *dipsaceus*

Teshale Assefa[1], Hailemichael Tesso[1], Venkatesha Perumal Ramachandran[1], Leta Guta[2], Taye B. Demissie[3], Japheth O. Ombito[3], Rajalakshmanan Eswaramoorthy[4], Yadessa Melaku[1]*

[1]Department of Applied Chemistry, Adama Science and Technology University, P.O. Box 1888, Adama, Ethiopia ;

[2]Department of Applied Biology, Adama Science and Technology University, P.O. Box 1888, Adama, Ethiopia ;

[3]Department of Chemistry, University of Botswana, P/Bag 00704, Gaborone, Botswana ;

[4]Department of Biomaterials, S aveetha Dental College and Hospitals, Saveetha Institute of Medical and Technical Sciences (SIMATS), Saveetha University, Chennai- 600 077, India

*Corresponding author : yadessa.melaku@astu.edu.et

We are hereby submitting supporting information for the manuscript entitled as "*In silico* Molecular Docking Analysis, Cytotoxicity and Antibacterial Activities of Constituents of Fruits of *Cucumis dipsaceus*. The supporting information includes the 2D and 3D interactions of the isolated compounds and standard drugs with the target proteins (Figures S 1-21).

Figure S 1: Binding interactions (3D & 2D) of compounds **3** with *E. coli* DNA gyrase (PDB ID: 6F86)

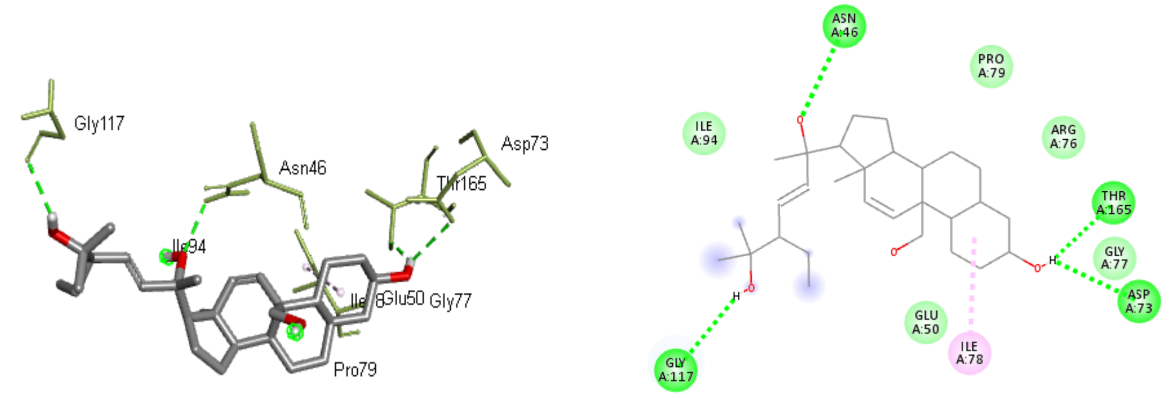

Figure S 2: Binding interactions (3D & 2D) of compounds **4** with *E. coli* DNA gyrase (PDB ID: 6F86)

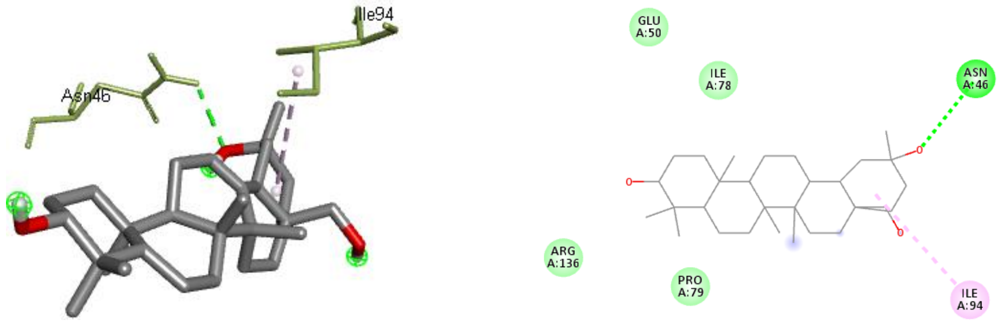

Figure S 3: Binding interactions (3D & 2D) of compounds **6** with *E. coli* DNA gyrase (PDB ID: 6F86)

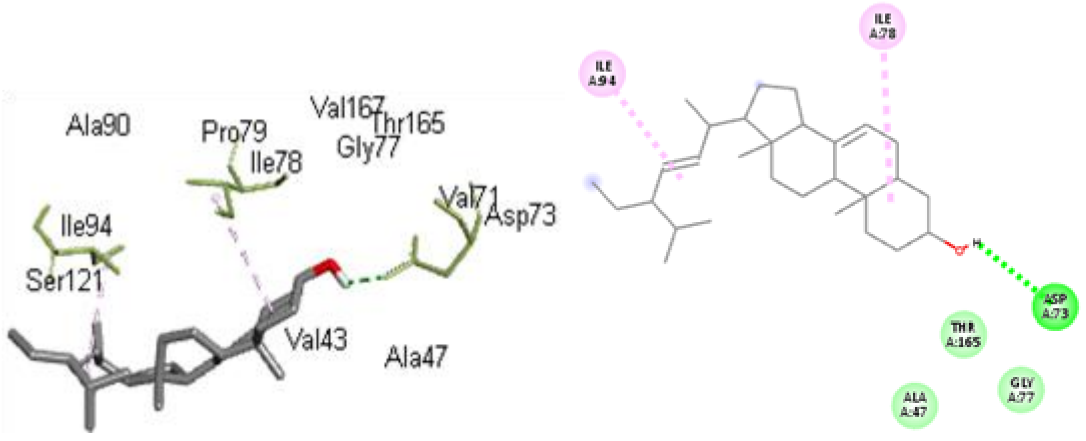

Figure S 4: Binding interactions (3D & 2D) of compounds **7** with *E. coli* DNA gyrase (PDB ID: 6F86)

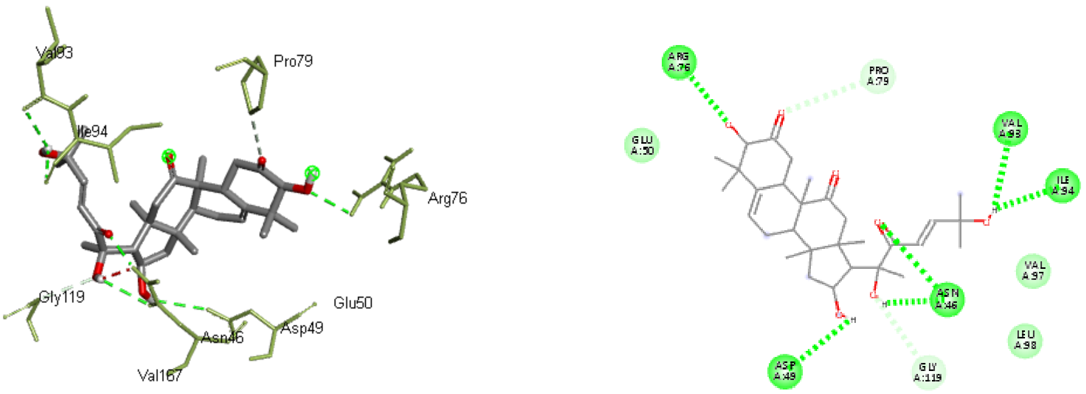

Figure S 5: Binding interactions (3D & 2D) of compounds **8** with *E. coli* DNA gyrase (PDB ID: 6F86)

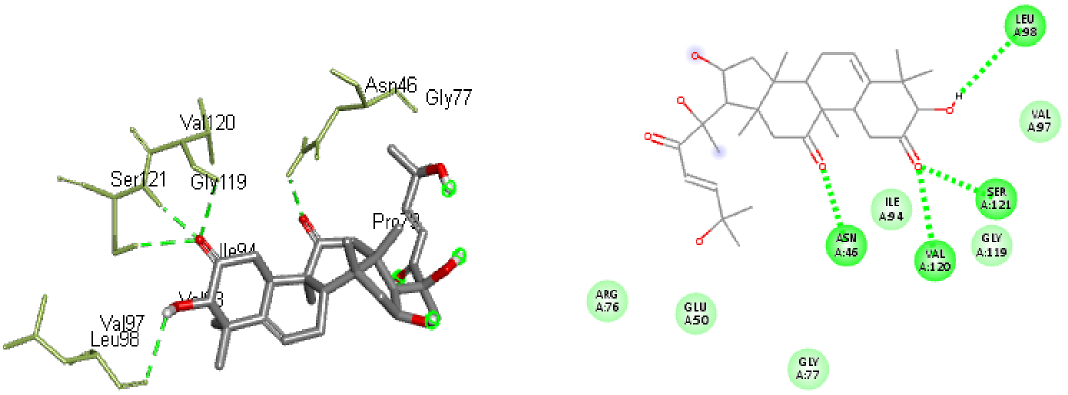

Figure S 6: Binding interactions (3D & 2D) of compounds **9** with *E. coli* DNA gyrase (PDB ID: 6F86)

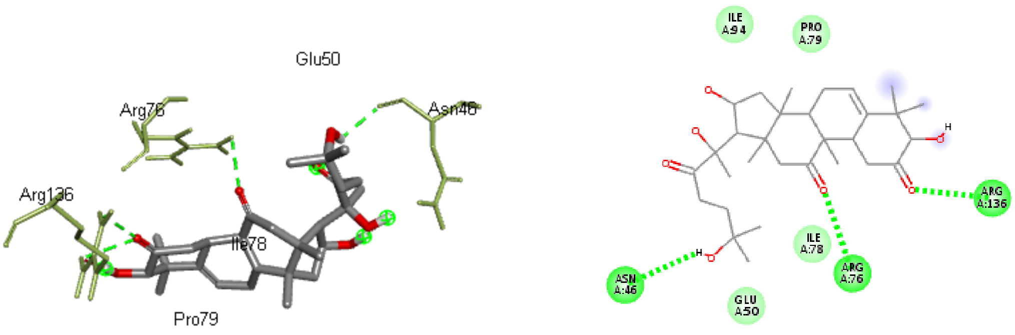

Figure S 7: Binding interactions (2D & 3D) of ciprofloxacin with *E. coli* DNA gyrase (PDB ID: 6F86)

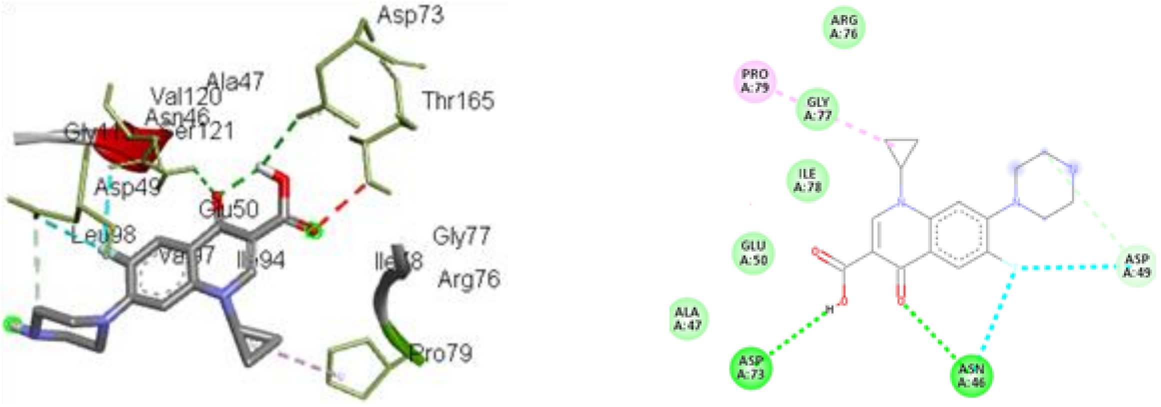

Figure S 8: Binding interactions (3D & 2D) of compounds **3** with S. *aurous* PK (PDB ID: 3T07)

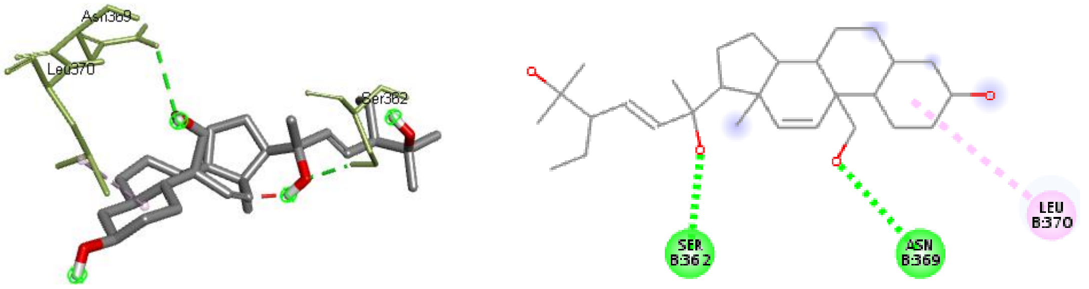

Figure S 9: Binding interactions (3D & 2D) of compounds **4** with S. *aurous* PK (PDB ID: 3T07)

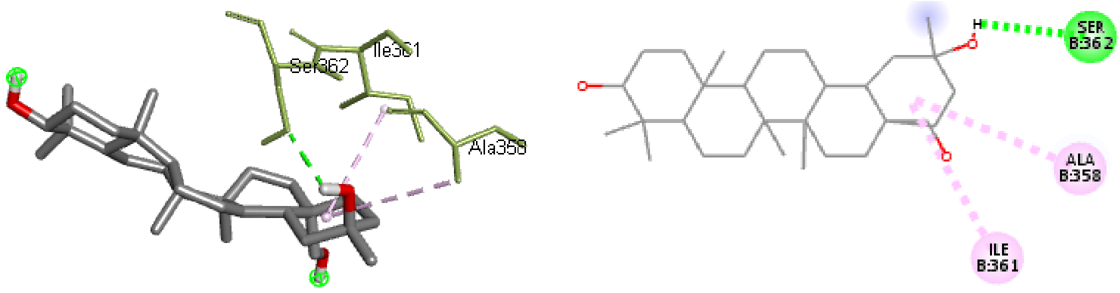

Figure S 10: Binding interactions (3D & 2D) of compounds **6** with S. *aurous* PK (PDB ID: 3T07)

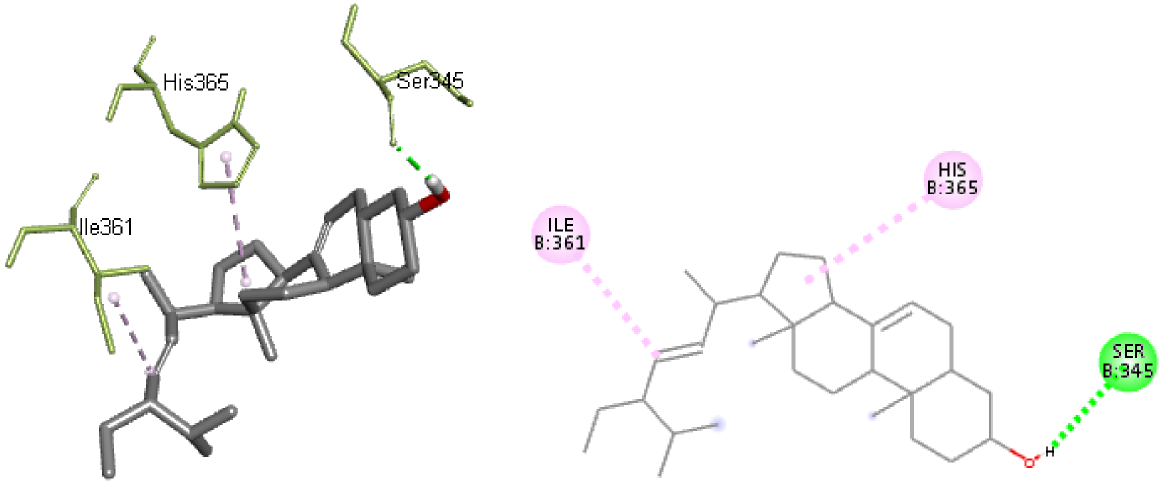

Figure S 11: Binding interactions (3D & 2D) of compounds 7 with S. *aurous* PK (PDB ID: 3T07)

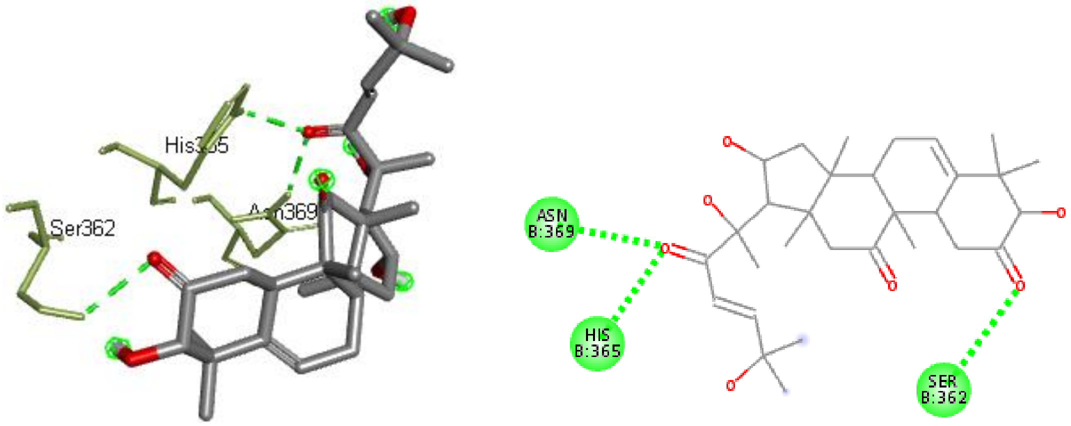

Figure S 12: Binding interactions (3D & 2D) of compounds **8** with S. *aurous* PK (PDB ID: 3T07)

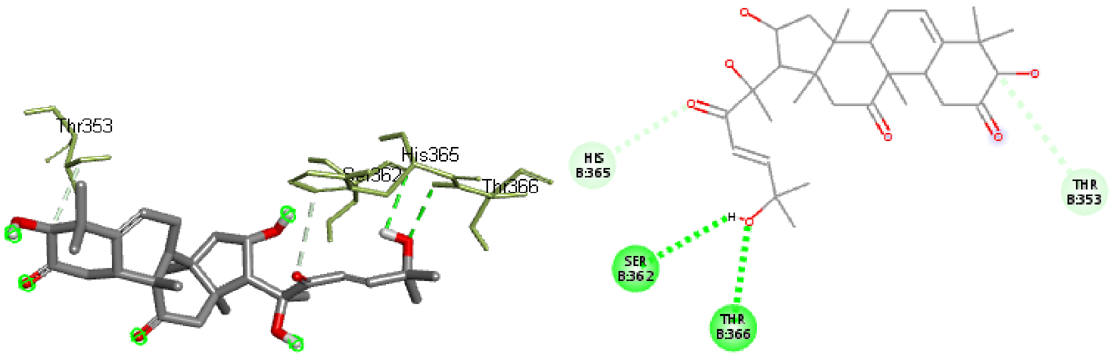

Figure S 13: Binding interactions (3D & 2D) of compounds **9** with S. *aurous* PK (PDB ID: 3T07)

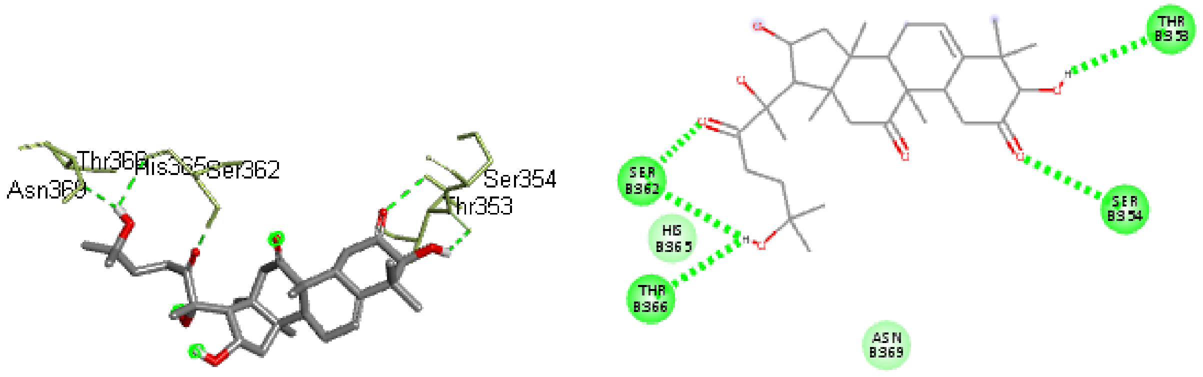

Figure S 14: Binding interactions (3D & 2D) of ciprofloxacin with S. *aurous* PK (PDB ID: 3T07)

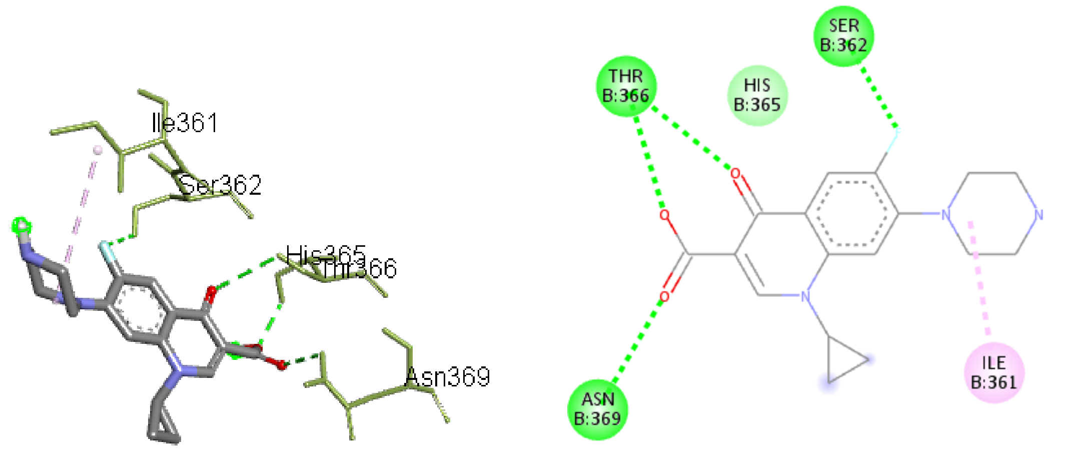

Figure S 15: Binding interactions (3D & 2D) of compounds **3** with human topoisomerase IIβ (PDB ID: 3QX3)

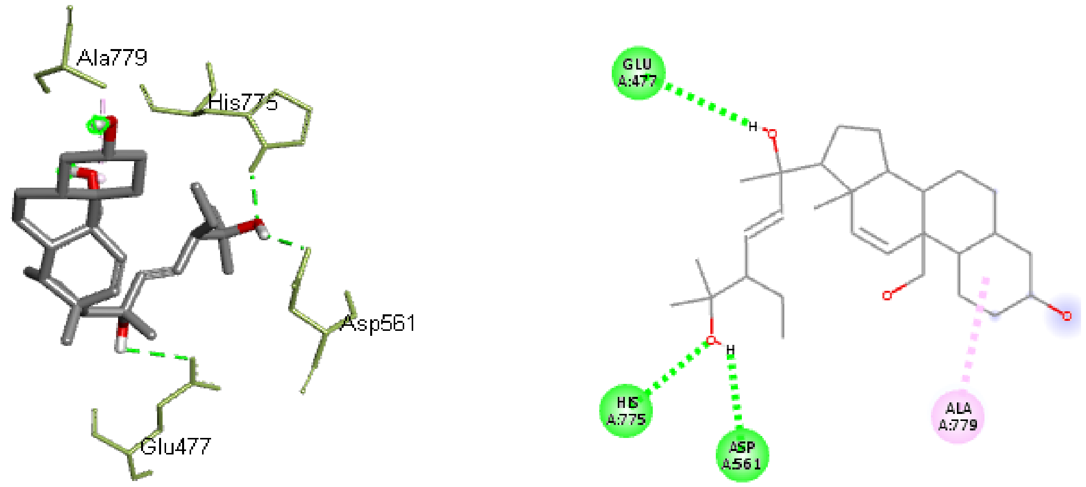

Figure S 16: Binding interactions (3D & 2D) of compounds **4** with human topoisomerase IIβ (PDB ID: 3QX3)

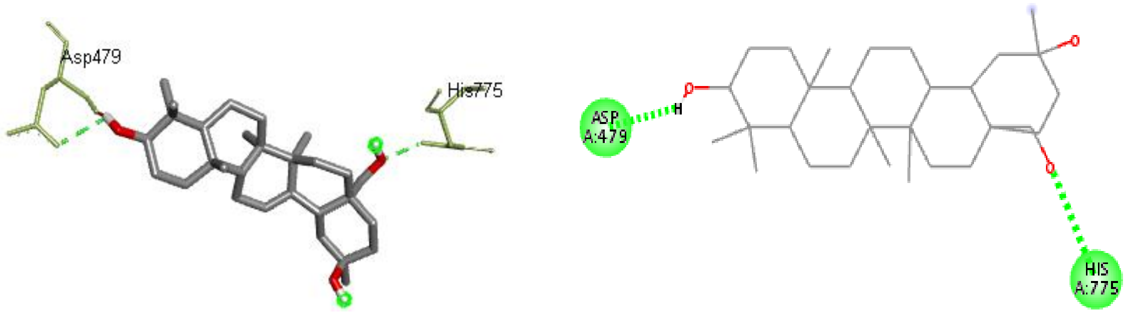

Figure S 17: Binding interactions (3D & 2D) of compounds **6** with human topoisomerase IIβ (PDB ID: 3QX3)

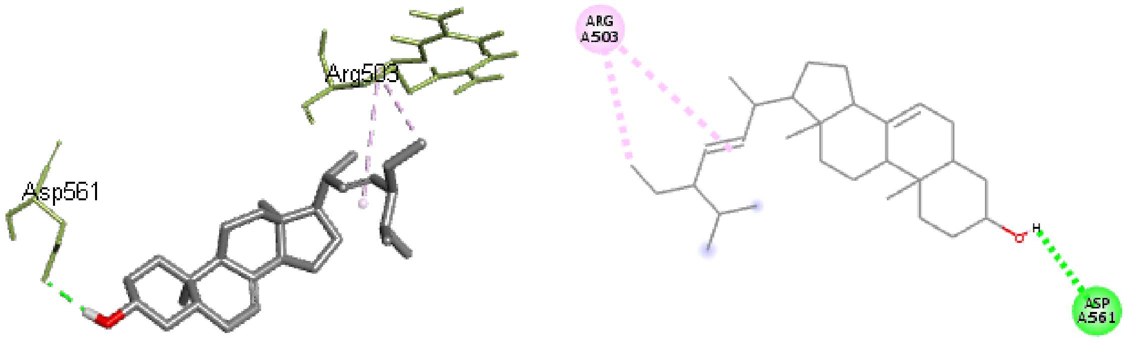

Figure S 18: Binding interactions (3D & 2D) of compounds 7 with human topoisomerase IIβ (PDB ID: 3QX3).

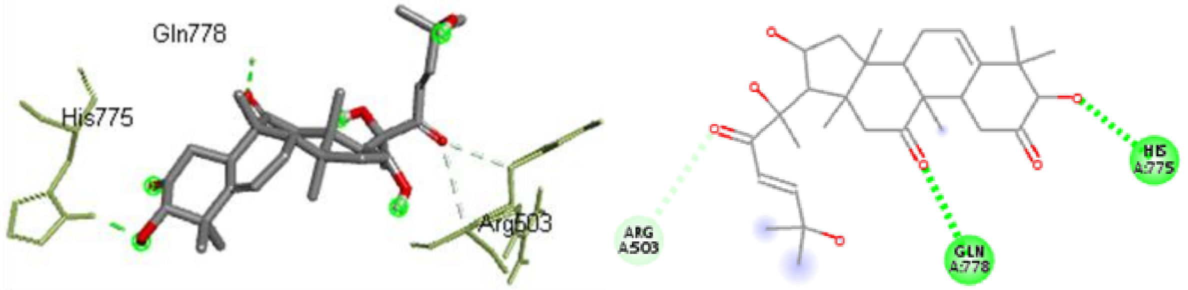

Figure S 19: Binding interactions (3D & 2D) of compounds **8** with human topoisomerase IIβ (PDB ID: 3QX3)

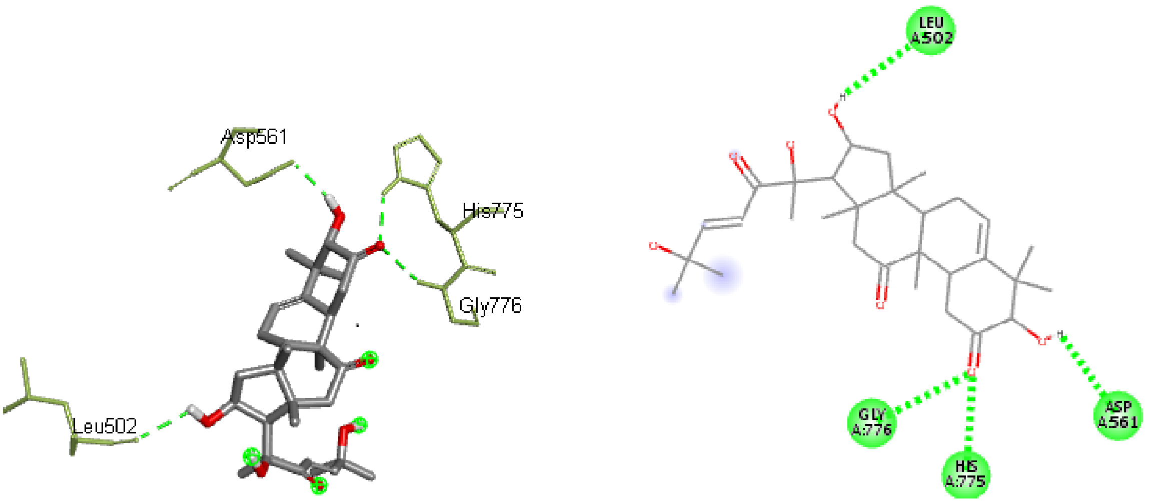

Figure S 20: Binding interactions (3D & 2D) of compounds **9** with human topoisomerase IIβ (PDB ID: 3QX3)

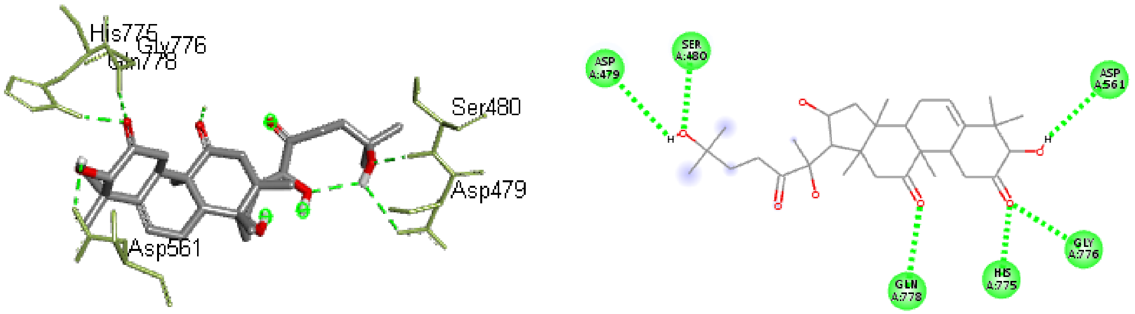

Figure S 21: Binding interactions (3D & 2D) of etoposide with human topoisomerase IIβ (PDB ID: 3QX3).

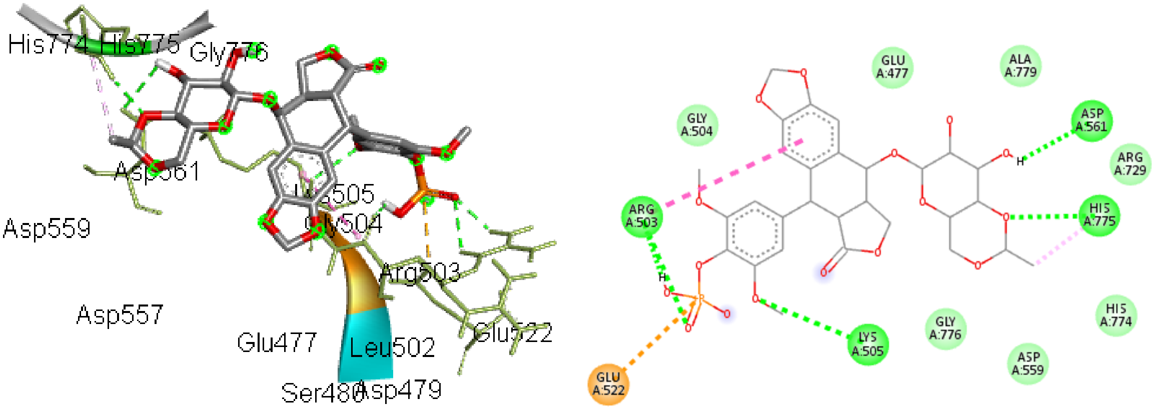

Supplement: Supplementary file 1 — ao3c08866_si_001.pdf [file ao3c08866_si_001.pdf]
